# Chicago Biological Society

**Published:** 1882-05

**Authors:** 


### ARTICLE VIII.

CHICAGO BIOLOGICAL SOCIETY. Stated Meeting, April 5, 1882. Dr. C. Fenger, President, in the Chair.

The minutes of the last meeting were read by Dr. Lester Curtis, in the absence of the Secretary, Dr. Roswell Park, who has gone to spend the summer in Europe.

The paper of the evening was read by Mr. E. B. Stuart, associate editor of *The Druggist*, and had the title: "The Nature and the Affinities of Certain Submerged Fungoid Growths." (Published in this number of the JOURNAL.)

Discussion.—Dr. Plim. S. Hayes asked the lecturer whether boracic acid would prevent the growth of fungi in alkaloidal solutions, and was answered in the affirmative.

Dr. C. Fenger remembered a proprietary article called *asepsine*, which had been sold extensively for destroying molds, and which contained nothing but boracic acid. Dr. Fenger said that the subject of organic germs was acquiring an important notoriety of late. He classed the forms known as aspergillus and mycelium among fungi.

As to their relation to pathological processes, but little yet was known. The first of them found to be harmful was *oidium albicans*, which causes in children the disease called thrush or muguet. This fungus was thought harmless at first, but it was subsequently found that in rare cases it had extended to and covered the walls of the stomach. The fact was also noticed that sometimes molds had been found growing in the external ear of persons who had slept near damp walls, and the fungus was supposed to have come from the walls.

The next form investigated was a dangerous one, and caused the disease called *actino-mycosis*, which resembles pyæmia. It was supposed to start its growth in some hollow teeth, thence extending in small abscesses to the submaxillary and other glands, and from there extending to and starting abscesses in the various parts of the body, causing death through pyæmia. These fungi were visible to the naked eye, the pus from the abscesses containing small grains resembling seeds. A plain mycelium was seen under the microscope. The theory was, as to those cases, that they always started from hollow teeth, though but little was known about them. These were all the fungi known to have multiplied in the human body.

Of late, however, experiments had been made with various fungi, the *Penicilium* and others, and, after they had been cultivated in certain manners, they were injected into the lower animals, dogs and rats, and in a great many cases the animals died in two, three, or five days. At the autopsy, small visible plaques were found in the kidneys, and under the microscope the mycelium of the fungus was always present.

However, these experiments were not perfect, and unimpeachable, for it had not always been possible to exclude any other form of fungi from those under observation.

Dr. Fenger should not wonder if the fungi studied by Mr. Stuart, under some circumstances, might be injected into the blood and there multiply. Although there were no cases on record pointing to that view, this should not stop researches, which were so meagre on that subject. It was a matter of some importance whether by the use of muddy solutions of alkaloids with a hypodermic syringe, some fungi would not be injected into the blood of patients.

In cases where patients died after the use of hypodermic injections, it would be well to look for plaques of mycelium in the kidneys and other viscera.

Mr. Stuart said that, as to the growth of fungi (saprolignæ) on animals, it was not infrequent. Flies were attacked that way, and so were certain fishes. These fungi might grow in the blood of other animals also, especially the warm-blooded, as a higher temperature was specially favorable to their growth. He had noticed that the spores of certain fungi, furnished with two filaments, had an individual motion, and sought the interior of tissues where to grow, as if they had been endowed with volition, and he looked upon them as possessing some intelligence, involving choice.

